# Acquired Type III Secretion System Determines Environmental Fitness of Epidemic *Vibrio parahaemolyticus* in the Interaction with Bacterivorous Protists

**DOI:** 10.1371/journal.pone.0020275

**Published:** 2011-05-23

**Authors:** Carsten Matz, Bianka Nouri, Linda McCarter, Jaime Martinez-Urtaza

**Affiliations:** 1 Helmholtz Centre for Infection Research, Braunschweig, Germany; 2 Microbiology Department, University of Iowa, Iowa City, Iowa, United States of America; 3 Instituto de Acuicultura, Universidad de Santiago de Compostela, Santiago de Compostela, Spain; University of Wisconsin-Milwaukee, United States of America

## Abstract

Genome analyses of marine microbial communities have revealed the widespread occurrence of genomic islands (GIs), many of which encode for protein secretion machineries described in the context of bacteria-eukaryote interactions. Yet experimental support for the specific roles of such GIs in aquatic community interactions remains scarce. Here, we test for the contribution of type III secretion systems (T3SS) to the environmental fitness of epidemic *Vibrio parahaemolyticus*. Comparisons of *V. parahaemolyticus* wild types and T3SS-defective mutants demonstrate that the T3SS encoded on genome island VPaI-7 (T3SS-2) promotes survival of *V. parahaemolyticus* in the interaction with diverse protist taxa. Enhanced persistence was found to be due to T3SS-2 mediated cytotoxicity and facultative parasitism of *V. parahaemolyticus* on coexisting protists. Growth in the presence of bacterivorous protists and the T3SS-2 genotype showed a strong correlation across environmental and clinical isolates of *V. parahaemolyticus*. Short-term microcosm experiments provide evidence that protistan hosts facilitate the invasion of T3SS-2 positive *V. parahaemolyticus* into a coastal plankton community, and that water temperature and productivity further promote enhanced survival of T3SS-2 positive *V. parahaemolyticus*. This study is the first to describe the fitness advantage of GI-encoded functions in a microbial food web, which may provide a mechanistic explanation for the global spread and the seasonal dynamics of *V. parahaemolyticus* pathotypes, including the pandemic serotype cluster O3:K6, in aquatic environments.

## Introduction

Horizontal gene transfer and the acquisition of foreign DNA is a fundamental process in the ecology and evolution of most bacterial species. Analysis of the increasing number of bacterial genome sequences has revealed considerable genomic variation among closely related strains, which is concentrated in mobile genetic elements, the genome islands (GIs) [1], [2]. GIs have been classified based on the different functions they encode, which include metabolic islands, degradation islands, resistance islands, symbiosis islands, and pathogenicity islands [3], [4]. The acquisition of GIs allows bacteria to instantly obtain a range of genetic traits that may increase fitness under different environmental conditions. The cyanobacterium *Prochlorococcus* and γ-proteobacteria of the genus *Vibrio* have become important models for the study of island genes, dynamics of genome diversity and niche partitioning in the marine environment [5], [6]. Despite initial convincing evidence for the adaptive significance of island genes among environmental bacteria, the precise functions of their products have rarely been characterized and their potential role in the evolution of independent bacterial lineages remains poorly understood.

*Vibrio parahaemolyticus* is a ubiquitous member of natural bacterioplankton communities in marine and estuarine environments [7], [8]. With one of the largest *Vibrio* genomes [9], *V. parahaemolyticus* is likely one of the most ecologically versatile. While many isolates of *V. parahaemolyticus* are considered non-pathogenic, infections among marine wildlife and humans have increased globally in the last years leading to the bacterium's classification as a newly emerging pathogen [10], [11], [12]. In 1996 the first appearance of a pandemic clone of *V. parahaemolyticus* occurred, a new O3:K6 serotype strain that has now been identified worldwide as the major cause of acute seafood-borne gastroenteritis [13], [14], [15], [16]. Genome sequence analyses show that there is considerable genomic flux in this species and that the new highly virulent clone arose from an O3:K6 isolate that acquired at least seven novel *V. parahaemolyticus* island regions (VPaI-1 to 7) [9], [17], [18]. Among these genome islands, VPaI-7 (VPA1312–VPA1396), an 81 kb region present on chromosome II, is considered the most relevant for pathogenicity to humans as it locates an additional set of type III secretion system genes (T3SS-2) and two copies of *tdh* genes encoding the thermostable direct hemolysin TDH [19]. Both sets of genes are key virulence factors in human gastroenteritis causing cytotoxicity and enterotoxicity, respectively [20], [21]. The occurrence of VPaI-7 in environmental strains suggests that T3SS-2 is not exclusively linked to enterotoxicity in humans but also contributes to the fitness and pandemic spread of *V. parahaemolyticus* pathotypes in marine environments [14], [11], [22]. The rapid global dissemination and recurring epidemics of *V. parahaemolyticus* raise the question as to whether VPaI-7 encoded T3SS-2 and TDH contribute to the environmental fitness of *V. parahaemolyticus* epidemic pathotypes.

As members of the natural bacterioplankton community, *Vibrio* spp. are an integral part of marine plankton communities. Like *V. cholerae*, *V. parahaemolyticus* has been found to associate with plankton organisms and suspended particulates as well as to be free-living in water [23], [24]. While chitinous zooplankton may primarily serve as recombination niche and transmission vehicle [25], [26], [27], protist populations appear to be hotspots of dissolved organic matter (DOM) supplemented growth or responsible for grazing-driven suppression and selection [28], [29], [30]. Hence, life of *V. parahaemolyticus* in association with auto- and heterotrophic protists suggests the possibility that the protein secretion machinery encoded on genome island VPaI-7 may bear specific functions in the interaction with aquatic protists.

In an initial effort to determine the potential roles of VPaI-7 encoded phenotypes in planktonic food webs, we conducted laboratory experiments in which wildtype strains and mutants defective in VPaI-7 encoded phenotypes were exposed to a range of bacterivorous protists. Specifically, we tested the contribution of T3SS-2 to fitness parameters relevant for the seasonal accumulation and global dissemination of *V. parahaemolyticus*, such as bacterial growth, persistence and invasion of microbial communities.

## Materials and Methods

### Organisms and culture conditions

The *V. parahaemolyticus* strains and mutants used in this study are listed in Table 1. The pre-1995 wild type LM5312 originates from coastal waters off Bangladesh (Robert Belas, personal communication), and the post-1995 wild type RIMD2210633 had been isolated during a gastroenteritis epidemic in Japan [31]. In addition, twenty-seven *V. parahaemolyticus* isolates were included in this analysis, eleven from environmental and sixteen from clinical sources (Table S1). These isolates were collected from Asia, Europe, South America, and the United States between 1951 and 2005, six of which belong to the pandemic pathotype O3:K6. Strains were routinely grown in Luria-Bertani (LB) broth with 3% NaCl or on equivalent agar plates and, prior to the experiments, in 40% artificial seawater (ASW) containing 10% LB broth.

**Table 1:** Bacterial strains.

Strain	Genotype or description	Source or reference
Strains		
*V. parahaemolyticus*		
LM5312	Wild type O4:K8	[34]
LM5674	*ΔopaR1*	[61]
LM7026	*Δvpa1342*::Kan^r^ *ΔopaR1*	This study
LM7035	*Δvp1672*::Cam^r^ *ΔopaR1*	This study
RIMD2210633	Wild type O3:K6	[31]
POR1	*ΔtdhAS*	[21]
POR2	*ΔtdhAS ΔvcrD1*	[21]
POR3	*ΔtdhAS ΔvcrD2*	[21]

The protists used in this study were marine and freshwater representatives of three common taxonomic groups: the flagellates *Cafeteria roenbergensis*, *Rhynchomonas nasuta* and *Ochromonas* sp., the ciliates *Tetrahymena* sp. and *Strombidium* sp., and the amoebae *Acanthamoeba castellanii* ATCC 30234 and *Dictyostelium discoideum*. Cultures of *R. nasuta*, *C. roenbergensis*, *Ochromonas* sp., *Tetrahymena* sp., *A. castellanii* and *D. discoideum* were axenic and maintained as described previously [32], [29], [33]. For all experiments, protists were taken from 5-day-old stock cultures.

### Construction of deletion mutants of *V. parahaemolyticus*

Deletion mutants with defects in T3SS-1 and T3SS-2 were made in *V. parahaemolyticus* LM5674. LM5674 was derived from BB22 [34]; it contains an 85-bp deletion in the upstream and N-terminal coding region of *opaR* (Δ*opaR1*) [35]. Deletion-insertion mutations were introduced into each of the T3SS genes encoding the EscR-type orthologs to make Δ*vp1672*::Cam^r^ (418 bp deletion) and Δ*vpa1342*::Kan^r^ (263 bp deletion). The mutations were constructed on *V. parahaemolyticus*-derived cosmids carrying T3SS genes by using a λ Red recombinase system in *Escherichia coli* [36]. The deletion-insertion constructs were then conjugated into *V. parahaemolyticus* and subsequently transferred to the chromosome of strain LM5674 by allelic exchange [37]. The allelic replacements were confirmed by Southern blot analysis.

### Grazing and cytotoxicity experiments

Experiments testing the survival of *V. parahaemolyticus* in the presence of bacterivorous protists were performed in 24-well tissue culture plates. Overnight cultures of the *V. parahaemolyticus* strains were diluted to 10^6^ cells mL^−1^ in 40% ASW, transferred into tissue culture plates and incubated at room temperature with moderate shaking. Subsequently, protists were added at a final concentration of 1×10^3^ cells mL^−1^. Numbers of flagellates and bacteria were followed over seven days. Generally each treatment was run in replicate wells of four.

### Plankton community experiment

A surface water sample (0–0.5 m) was collected from the Baltic Sea (Dahme Pier, Germany) in August 2007 with a thoroughly rinsed and autoclaved 5-L polycarbonate bottle (Nalgene). The water was stored at *in situ* temperature until filtration. Water was filtered through pre-rinsed glass-fibre filters with a nominal pore size of 1.0 mm (Whatman GF/C). For the microcosm experiment, 450 mL of filtered seawater was distributed into each of eight acid-cleaned 500-mL glass flasks. The protist-free treatment received seawater, which was first passed through a 0.8-µm pore-size membrane filter to remove nano- and microplankton while retaining the natural bacterial community. Prior to the experiments, a loopful of each *V. parahaemolyticus* strain was suspended in 150 mL of 40% ASW amended with 0.1% (wt/vol) tryptone and incubated at room temperature (23±1°C) overnight. Cells were then harvested by centrifugation and the remaining pellet was suspended in 40% ASW. This washing process was repeated four times in order to prevent the carryover of medium nutrients. Finally, the pellet was suspended in 40% ASW for subsequent inoculation of microcosm flasks.

Protist-free microcosm and protist microcosm were amended with either *V. parahaemolyticus* T3SS-2 positive RIMD2210633 or T3SS-2 negative POR3. *V. parahaemolyticus* was added to an average initial concentration of 10^5^ cells mL^−1^. Each of the four different treatments was performed in duplicate, yielding a total of eight experimental microcosms. Additional controls contained only the natural plankton community. Microcosms were incubated for five days at 22±1°C (under ambient laboratory light conditions) and moderate mixing on a rotary shaker. Each microcosm was sampled daily for direct cell counts of immunofluorescent *V. parahaemolyticus* and photoautrophic and heterotrophic protists.

### Temperature and productivity experiment

To study the effect of elevated temperature and productivity on the outcome of *Vibrio*-protist interactions, we exposed the T3SS-positive *V. parahaemolyticus* RIMD2210633 and the T3SS-negative mutant POR3 to the thermo- and osmotolerant ciliate *T. pyriformis*. Cocultures of *V. parahaemolyticus* and *T. pyriformis* were incubated in 24-well tissue culture plates containing 40% ASW, with initial bacteria and protist concentrations of 1×10^5^ cells mL^−1^ and 1×10^3^ cells mL^−1^, respectively. Temperature treatments were supplemented with 2 mg tryptone mL^−1^ and were incubated at 16, 23 and 30°C, respectively. Productivity effects were tested by adding tryptone to a final concentration of 2, 10, and 20 mg mL^−1^, respectively and were incubated at 23°C. Each treatment was run in replicate wells of four for 5 days.

### Detection of tdh and T3SS genes

Polymerase chain reaction assays were performed to test for the presence of T3SS-2 positive *V. parahaemolyticus* in coastal bacterioplankton samples. After enrichment in alkaline peptone water (1% (wt/vol) peptone, 1% (wt/vol) NaCl; pH 8.2), seawater aliquots showing bacterial growth were analyzed by PCR targeting *tdh* and T3SS-2 genes. Primers and amplification conditions were those reported previously [38], [39].

### Enumeration of bacteria and protists

Numbers of *V. parahaemolyticus* and protists were determined by means of epifluorescence microscopy by using the nonspecific DNA-stain DAPI after adding 4% glutaraldehyde to each well to get a final concentration of 2%. In the plankton community experiment, *V. parahaemolyticus* was detected by immunofluorescence microscopy employing mouse polyclonal antiserum (BioGenes, Berlin, Germany) that was raised against strain RIMD2210633 and a Cy3-conjugated goat anti-mouse immunoglobulin G (Jackson ImmunoResearch Laboratories) [32]. In all experiments, cell densities of *V. parahaemolyticus* were also assessed by plating serial dilutions on LB and TCBS agar.

### Virulotyping of *V. parahaemolyticus* isolates

We determined the pathogenic potential of eleven environmental and sixteen clinical isolates of *V. parahaemolyticus* (Table S1) by analysing bacterial growth on amoeba-seeded agar plates [40]. Briefly, six-day-old cultures of *A. castellanii* were harvested by centrifugation (500 g, 5 min) and resuspended in 40% ASW to give a final concentration of 2×10^6^ cells mL^−1^. Seeded agar plates were prepared by spreading 1.5 mL of the amoeba concentrate onto LB agar and allowed to dry for 1–2 h in a biohazard hood. Stationary-phase cultures of *V. parahaemolyticus* strains were adjusted to an identical OD600 and serially diluted in 96-well microtiter dish. Approximately 3 µL of each dilution step were spotted onto amoeba-seeded agar plates by using a 96-pin replicator. Agar plates were prepared in replicates of four and incubated for four days at 22°C. Minimum survival concentrations were determined for each *V. parahaemolyticus* strain from the highest dilution of *V. parahaemolyticus* that gives rise to robust colonies in the presence of *A. castellanii*.

### Statistical analysis

Changes in bacterial and protist numbers over time were tested for significance with repeated measures ANOVA. Survival percentages were arcsine-square root transformed. Pairwise comparisons of means were done by Student's t-tests.

## Results

### Genome island VPaI-7 promotes survival of *V. parahaemolyticus* in the interaction with marine protist

Protists can function as microbial predators, competitors or hosts, and thus play important roles in the adaptation and evolution of aquatic bacteria. We studied the contribution of genome island VPaI-7, which encodes the accessory type III secretion system T3SS-2 and the temperature-dependent hemolysin TDH, to growth and survival of *V. parahaemolyticus* in the presence of the marine nanoflagellate *C. roenbergensis*. We compared two marine isolates of *V. parahaemolyticus* (VPaI-7 negative strain PM220 and VPaI-7 positive LM5674) with the pandemic strain RIMD2210633 harboring VPaI-7. VPaI-7 mediated effects were tested by using isogenic mutants of *V. parahaemolyticus* LM5674 and RIMD2210633 (Table 1).

**Figure 1:**
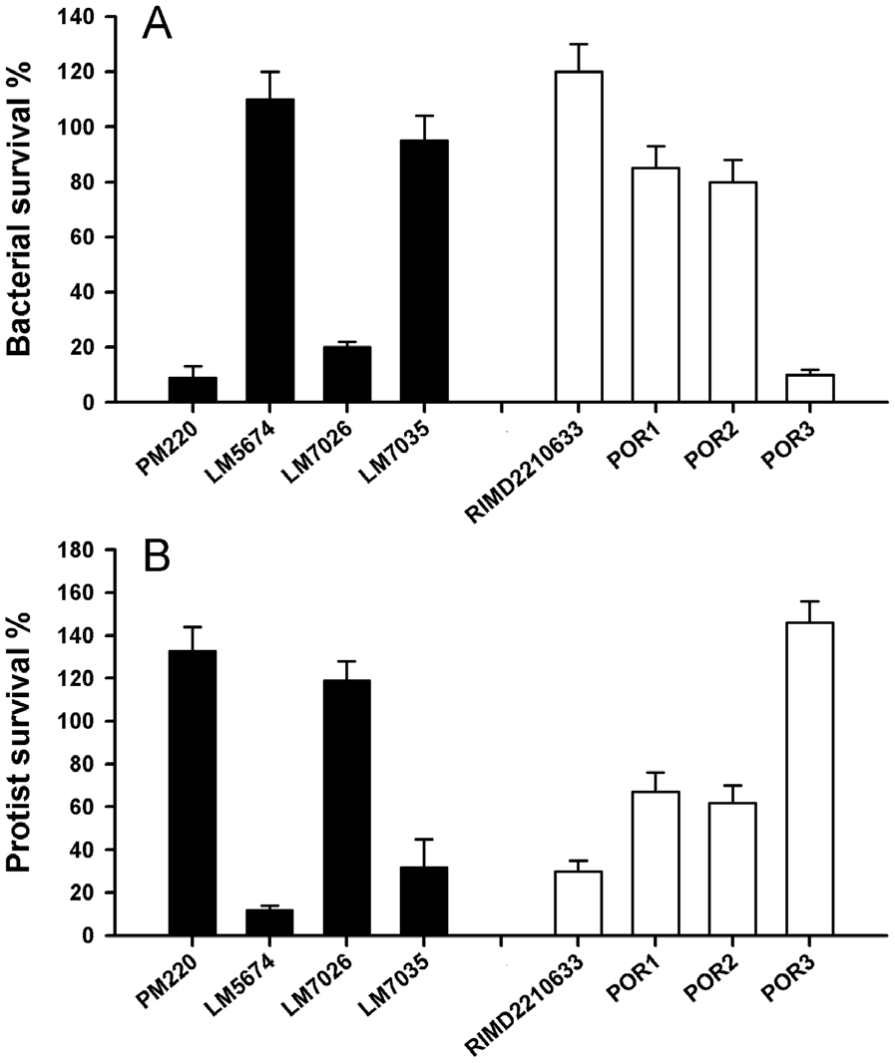
Accessory type III secretion system promotes survival of *V. parahaemolyticus*. Cell suspensions of environmental (filled bars) and clinical strains (open bars) of V. parahaemolyticus were co-cultured with the marine flagellate *C. roenbergensis*. Bacterial (A) and flagellate (B) survival are given as relative change of initial cell numbers after 24 h of co-incubation of *C. roenbergensis* with *V. parahaemolyticus* strains: the environmental isolates PM220 (*tdh−*/T3SS-2−) and LM5674 (*tdh+*/T3SS-2+) versus mutants defective in T3SS-2 (LM7026) and T3SS-1 (LM7035); and the clinical isolate RIMD2210633 (*tdh+*/T3SS-2+) versus mutants defective in TDH (POR1), T3SS-1 (POR2) and T3SS-2 (POR3). Error bars represent standard deviations (n = 4).

Cell suspensions of the VPaI-7 negative strain PM220, which represents the dominant genotype in marine environments, were rapidly eliminated and supported growth of the nanoflagellate *C. roenbergensis* (Figure 1). Abundances of the VPaI-7 positive environmental strain LM5674, however, remained stable in the three days of co-culture (Figure 1A). Successful survival of LM5674 coincided with a more than 85% reduction of flagellate cell numbers indicating protist-targeting cytotoxicity (Figure 1B). By contrast, the T3SS-2 defective mutant LM7026 was rapidly reduced by 80%, which also resulted in increasing numbers of *C. roenbergensis* relative to initial flagellate numbers. Notably, survival and cytotoxicity of the isogenic T3SS-1 mutant LM7035 were comparable to the effects observed for the wild-type strain LM5674 (P>0.05).

Similar observations were made for the post-1995 clinical isolate RIMD2210633 and derived mutants. The *tdhAS* double-deletion mutant POR1 showed significantly lower bacterial survival and significantly higher flagellate survival compared to the wild type (both P<0.001). POR2, carrying an additional deletion in *vcrD1* encoding an inner membrane protein of the T3SS-1, showed no significant change on bacterial and flagellate survival rates relative to the *tdhAS* double-deletion mutant POR1 (both P>0.05). While the cytotoxicity of T3SS-1 defective mutant was indistinguishable from that of the parent strain, the T3SS-2 defective mutant POR3, carrying a deletion in vcrD2, showed the most significant decrease in cytotoxicity (P<0.001). Besides the significantly stronger effect of T3SS-2 relative to the T3SS-1 (P<0.001), the strain comparison revealed that the temperature-dependent hemolysin TDH contributed to the survival of *V. parahaemolyticus*. Consistent for both the environmental and the clinical isolates, genome island VPaI-7 encoding a functional T3SS-2 and the temperature-dependent hemolysin TDH is a key determinant of cytotoxicity and survival for *V. parahaemolyticus* in the presence of the marine protist *C. roenbergensis*.

### Prey-to-parasite lifestyle switch of *V. parahaemolyticus* mediated by the accessory type III secretion system

**Figure 2:**
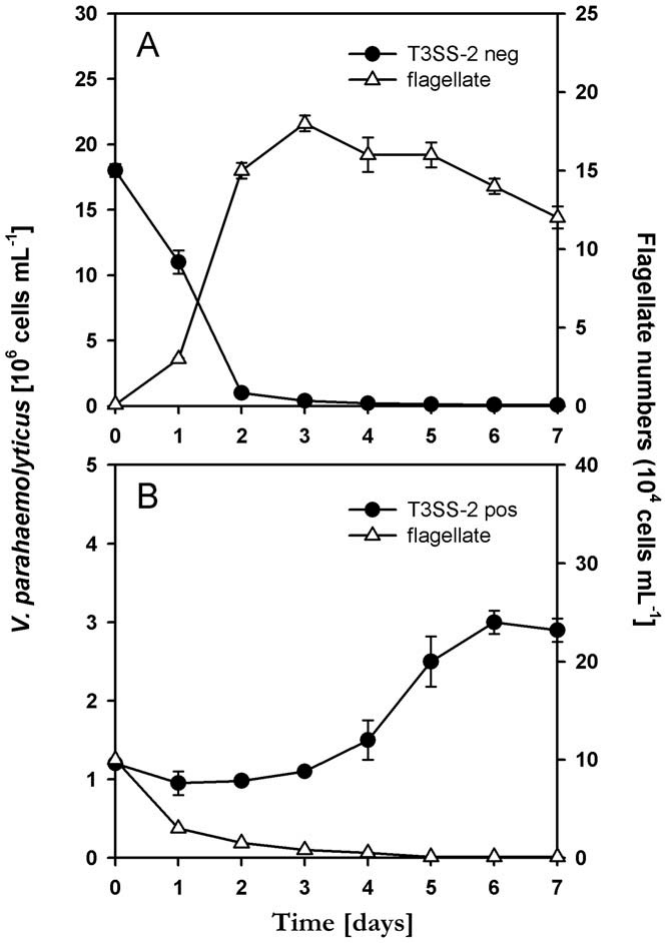
Prey-parasite lifestyle switch of *V. parahaemolyticus* as mediated by the accessory type III secretion system. Cell suspensions of the T3SS-2 deficient mutant POR3 (A) and the T3SS-2 positive wild type RIMD2210633 (B) were exposed to the flagellate *C. roenbergensis*. Flagellate numbers include only structurally intact cells and are given as means ± standard deviation (n = 4).

To study potential longer-term effects of T3SS-2 mediated cytotoxicity in *Vibrio*-protist interactions, we followed bacterial and flagellate cell numbers over a one-week-period by employing immunofluorescence microscopy. In treatments containing the T3SS-2 defective mutant POR3, *V. parahaemolyticus* was effectively reduced by *C. roenbergensis* below a concentration of 10^4^ cells mL^−1^ (Figure 2A). By contrast, populations of the T3SS-2 positive wild type RIMD2210633 remained unaffected due to the instant lysis of *C. roenbergensis* (Figure 2B). Direct cell counts revealed that upon the lytic break-down of *C. roenbergensis* populations *V. parahaemolyticus* increased significantly by about 3-fold relative to the initial cell number (P<0.001). CFU-based abundances of *V. parahaemolyticus* confirmed this finding as these numbers increased to the same extent (data not shown). Both datasets suggest that T3SS-2 positive *V. parahaemolyticus* is capable of killing protists and growing on the cell lysate, thus acting as facultative parasite for protist populations.

### T3SS-2 elicits cytotoxicity towards broad range of protist taxa

In natural microbial communities *V. parahaemolyticus* encounters a wide range of protist taxa, which raises the question as to how specific and effective T3SS-2 elicited pathogenicity is to different protistan hosts. We exposed seven protists representing major taxonomic phyla to suspensions of the T3SS-2 positive wild type RIMD2210633 and the T3SS-2 defective mutant POR3. The T3SS-2 positive wild type revealed acute cytotoxicity towards all seven protists tested (Table 2), resulting in a rapid decline in protist cell numbers within 48 h. Species-specific LT_50_ values, describing the time needed to kill 50% of the initial protist population, indicated the highest sensitivity of the three nanoflagellates *C. roenbergensis*, *R. nasuta* and *Ochromonas* sp. Protist numbers on the T3SS-2 defective mutant POR3, however, multiplied at rates comparable to those of *C. roenbergensis* depicted in Fig. 2A. By not considering LT_50_ values of *Strombidium* sp. due to the non-axenic state of the protist culture, we found a significant negative correlation between protist-specific susceptibility to the T3SS-2 positive wild type RIMD2210633 and protist cell size (R^2^ = 0.96, P<0.001).

**Table 2:** Susceptibility of diverse protist taxa to *V. parahaemolyticus* RIMD2210633.

Protist species	LT_50_ (h)
Flagellates	
*Cafeteria roenbergensis*	12±1.6
*Rhynchomonas nasuta*	13±0.8
*Ochromonas* sp.	15±1.4
Ciliates	
*Tetrahymena pyriformis*	32±2.4
*Strombidium* sp.^a^	59±1.9
Amoebae	
*Acanthamoeba castellanii*	25±1.7
*Dictyostelium discoideum*	19±1.7

LT_50_ values describe the time needed to kill 50% of the initial protist population.

Note that LT_50_ values on the T3SS-2 defective mutant POR3 could not be determined due to positive growth rates of all seven protists. Values are given as means ± standard deviation (n = 4).

a Note that cultures of *Strombidium* sp. were not axenic.

### Strain-specific fitness of *V. parahaemolyticus* correlates with T3SS-2 and tdh genotype

Based on our finding of T3SS-2 mediated survival in *V. parahaemolyticus* LM5674 and RIMD2210633, we extended our examinations to a total of 27 strains isolated from across the globe. These isolates included eleven environmental and sixteen clinical *V. parahaemolyticus* strains, six of which belong to the pandemic pathotype O3:K6 (Table S1). By using the amoeba-seeded agar plate assay, we analysed growth of *V. parahaemolyticus* strains in the presence of the amoeba *A. castellanii*. Stationary-phase cultures of *V. parahaemolyticus* strains were spotted in tenfold serial dilutions onto amoebae-seeded agar plates. Minimum survival concentrations were determined for each strain from the lowest cell concentration of *V. parahaemolyticus* to form robust colonies in the presence of *A. castellanii*.

**Figure 3:**
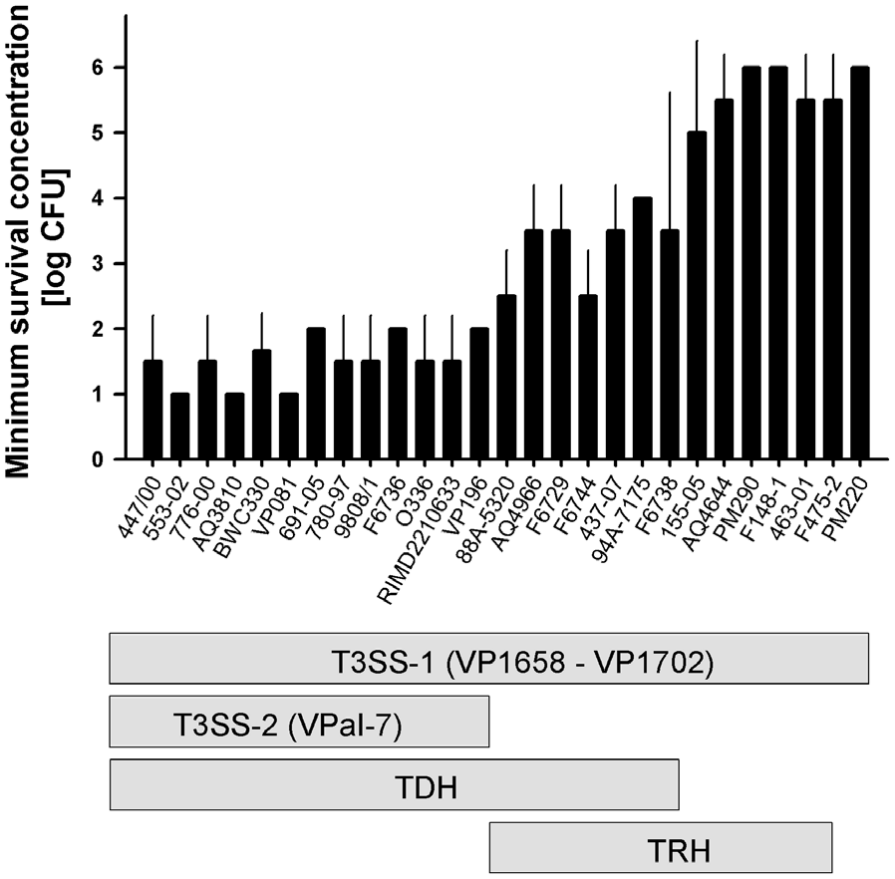
Antipredator effects correlate with T3SS-2 and TDH genotype in environmental and clinical strains of *V. parahaemolyticus*. Minimum survival concentrations of 27 strains of *V. parahaemolyticus* were calculated from the minimum bacterial concentration to form robust colony forming units on amoeba-seeded agar plates. Error bars indicate standard deviations of three repeated experiments.

Bacterial concentrations growing to robust colonies varied from 10^1^ to 10^6^ cells µL^−1^ (Figure 3). Strain-specific minimum survival concentrations were found to correlate with the presence/absence of TDH and T3SS-2 genes. Strains containing T3SS-2 had minimum survival concentrations as low as 10^1^ to 10^2^ cells µL^−1^, indicating that low cell densities are sufficient to grow and survive in the presence of *A. castellanii*. The highest cell densities to survive amoebal grazing were required by strains harboring neither T3SS-2 nor TDH genes; their minimum survival concentrations ranged from 10^5^ to 10^6^ cells µL^−1^. Strains containing tdh genes but the VPaI-8 encoded T3SS-trh instead of the T3SS-2 had minimum survival concentrations ranging from 10^2^ to 10^4^ cells µL^−1^. These findings are in full support of the data reported from our wild type versus mutant experiments as shown above.

### Invasion of T3SS-2 positive *V. parahaemolyticus* into coastal plankton

Epidemiological and biogeographical data indicate that T3SS-2 positive strains of *V. parahaemolyticus* have undergone a rapid global dissemination across marine and brackish habitats. To assess the contribution of T3SS-2 to the capacity of *V. parahaemolyticus* to invade local plankton communities, we added T3SS-2 positive wild type and T3SS-2 negative mutant to microcosms containing a naïve coastal plankton assemblage. PCR targeting *tdh* and T3SS-2 genes as well as immunofluorescence microscopy of plankton samples prior to the experiment revealed that the plankton community was free of T3SS-2 positive and immuno-positive *V. parahaemolyticus*, respectively.

**Figure 4:**
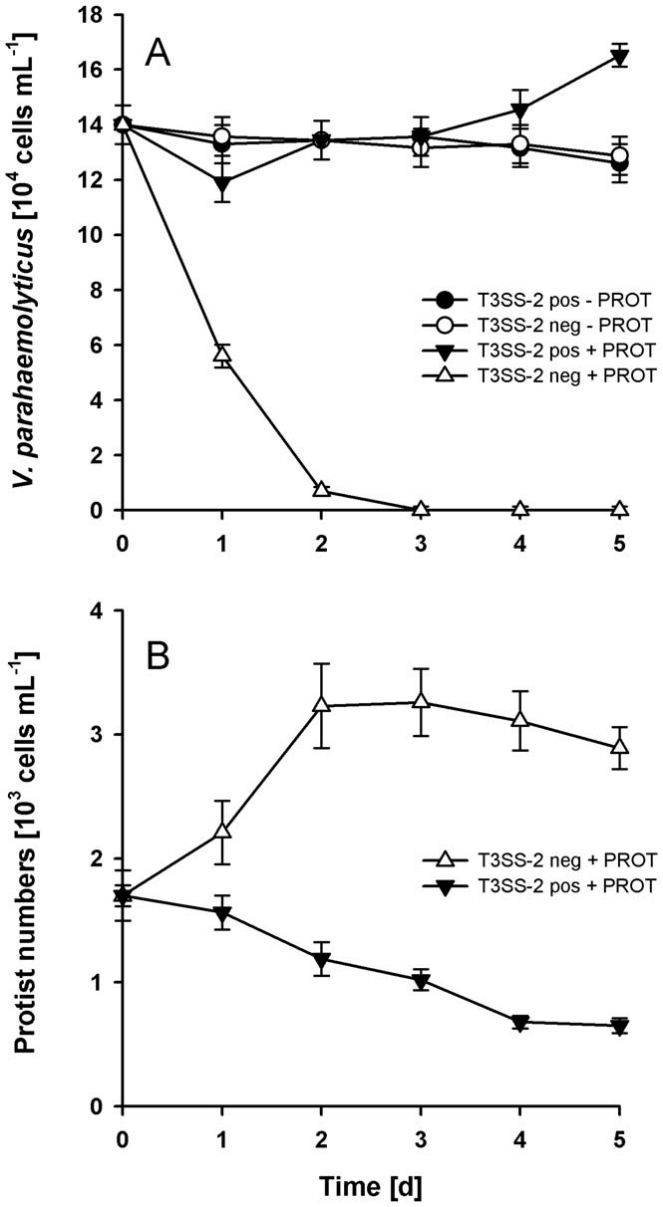
Invasion of T3SS-2 positive *V. parahaemolyticus* into coastal plankton community. Microcosms containing natural plankton communities were inoculated with the T3SS-2 positive wild type RIMD2210633 and the T3SS-2 deficient mutant POR3. (A) Survival of *V. parahaemolyticus* is given as relative change of initial cell numbers in the presence (+PROT) and absence (−PROT) of the natural protist community. (B) relative changes in total protist numbers are given for the protist microcosms. Error bars represent standard deviations (n = 4).

Treatments containing natural protist communities were compared with protist-free bacterioplankton treatments. In protist-free bacterioplankton (−PROT), populations of T3SS-2 positive wild type and T3SS-2 negative mutant decreased by about 10% over a five-day-period without significant differences between wild type and mutant (Figure 4A). In the presence of heterotrophic and photoautotrophic protists (+PROT), T3SS-2 negative *V. parahaemolyticus* showed a population decline by about 100-fold and was not able to establish *V. parahaemolyticus* populations at detectable densities. At the same time protists abundances increased from 1.7 to 3.2×10^3^ cells mL^−1^ (Figure 4B). Plankton communities inoculated with T3SS-2 positive *V. parahaemolyticus* were characterized by a stable *V. parahaemolyticus* population despite the presence of heterotrophic protists. However, numbers of heterotrophic and photoautotrophic protists in these treatments dropped by 59%, which coincided with an increase of T3SS-2 positive *V. parahaemolyticus* by 20% at day 5 (P<0.001, Figure 4A). These data confirm the cytotoxicity towards protists as described above and suggest a high capacity of the T3SS-2 positive genotype for biological invasion of natural plankton communities by using photoautotrophic and heterotrophic protist as facultative hosts.

### Temperature and productivity promote selective enrichment of T3SS-2 positive *V. parahaemolyticus*

**Figure 5:**
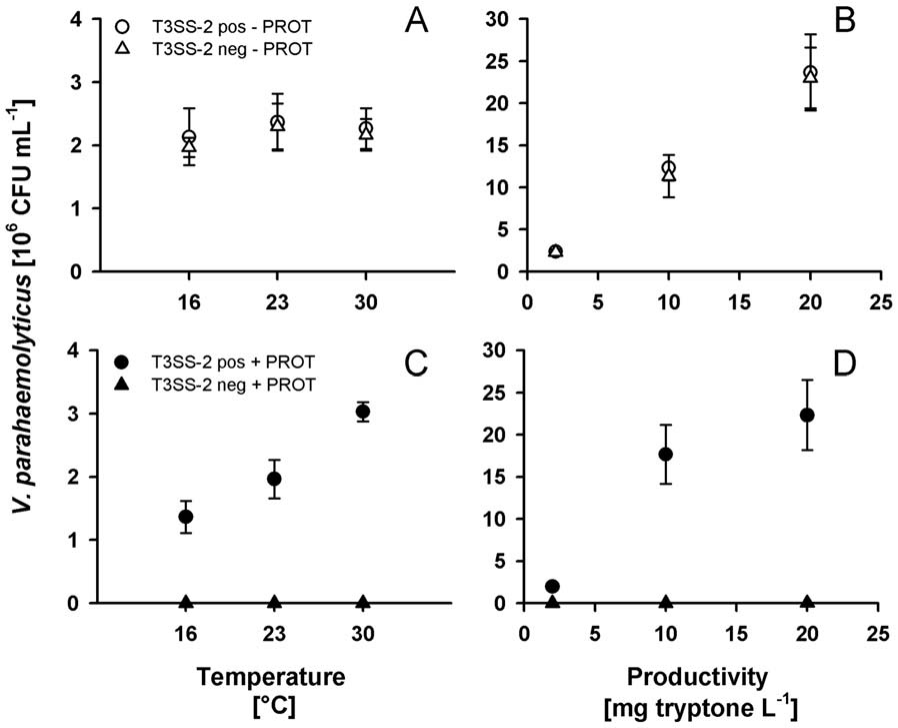
Effects of temperature and productivity on selective enrichment of T3SS-2 positive *V. parahaemolyticus*. Maximum bacterial abundances of the T3SS-2 positive wild type RIMD2210633 and the T3SS-2 deficient mutant POR3 were determined in the absence (open symbols) and the presence (closed symbols) of the ciliate *T. pyriformis*. Temperature effects (A and C) were tested using media containing 2 mg tryptone ml^−1^; productivity effects were examined at 23°C. Error bars represent standard deviations (n = 4).

Based on the fact that water temperature and eutrophication control *Vibrio* population dynamics in the field, we used the thermo- and osmotolerant ciliate *T. pyriformis* to study the effect of elevated temperature and nutrient richness on the outcome of *Vibrio*-protist interactions. Temperature was found to have no effect on maximum population densities of *V. parahaemolyticus* in the absence of *T. pyriformis* (Figure 5A). Both T3SS-2 positive wild type and T3SS-2 negative mutant performed equally well by responding to increased temperatures at similar rates (P>0.05). The presence of the ciliate *T. pyriformis* promoted growth of T3SS-2 positive relative to T3SS-2 negative *V. parahaemolyticus* (Figure 5C). However, maximum cell densities of T3SS-2 positive *V. parahaemolyticus* increased steadily from 16 to 30°C while growth of the T3SS-2 negative strain remained suppressed by the ciliate grazer. These significant temperature effects suggest population dynamics that are based on temperature-dependent growth or virulence of T3SS-2 positive *V. parahaemolyticus* and/or temperature-dependent clearance rates of *T. pyriformis*.

Similar to increasing temperature, elevated nutrient concentrations in the absence of *T. pyriformis* did not reveal significant differences between the maximum population densities of T3SS-2 positive and T3SS-2 negative *V. parahaemolyticus* (Figure 5B, P>0.05); tryptone enrichment resulted in increasing population densities for both strains. Ciliate grazing suppressed population growth of the T3SS-2 negative mutant completely while population densities of the T3SS-2 positive wild type were not negatively affected (Figure 5D). Interestingly, population densities of T3SS-2 positive *V. parahaemolyticus* did not show a significant increase from the intermediate to the highest productivity level (P>0.05). Taken together, fitness of *V. parahaemolyticus* in the presence of protists not only is strictly dependent upon T3SS-2 but further enhanced by elevated temperatures and productivity levels.

## Discussion

As environmental pathogens like *V. parahaemolyticus* are integral members of natural microbial communities, it is anticipated that some virulence factors studied in the context of human disease may have their functional origin in the interaction with coexisting eukaryotes. Toxins secreted by the *V. parahaemolyticus* T3SS are suspected to have profound effects on the progression and severity of gastroenteritis in humans [20], [21], [41]. Yet the pressures driving evolution of these toxins have been unclear, because humans are predominantly an accidental host of *V. parahaemolyticus*. The present findings suggest that the natural targets of one of the bacterium's T3SS may be environmental protists.

Our experiments demonstrate that the T3SS encoded on genome island VPaI-7 confers *V. parahaemolyticus* with a fitness advantage in the interaction with aquatic protists. T3SS are found in a wide range of γ-proteobacteria, especially in animal and plant pathogens or symbionts [42], [43]. Although the function of the T3SS as a generalized protein translocation apparatus is conserved in pathogens and mutualists, the precise role of any given T3SS is defined by the effector proteins that are translocated to the eukaryotic host cell. Our recent studies on the opportunistic pathogen *Pseudomonas aeruginosa* revealed that T3SS can play a central role in bacteria–protist interactions [44]. Studying co-cultures of *P. aeruginosa* and the amoeba *A. castellanii*, we found that *P. aeruginosa* rapidly colonized and killed coexisting amoebae. Analysis of the amoeba-induced transcriptome of *P. aeruginosa* indicated the expression of T3SS genes upon direct contact with amoebae. A comparison of mutants with specific defects in the T3SS demonstrated the use of the secretion apparatus and the effectors ExoU, ExoS, and ExoT in the killing process, in which ExoU had the greatest impact.

Some effector proteins secreted by *V. parahaemolyticus* T3SS-2 have recently been identified. VopA/P (VPA1346) is an acetyltransferase that inhibits ATP binding by acetylating the catalytic loop of mitogen-activated protein kinase kinases and shares about 55% similarity with the YopJ-like proteins from *Yersinia* and *Salmonella* [45]. VopC (VPA1321) is homologous to *Escherichia coli* cytotoxic necrotizing factor (38% identity) [46]. VopL (VPA1370) induces formation of actin stress fibers and manipulates actin assembly of HeLa cells [47]. VopT (VPA1327) has ADP-ribosyltransferase (ADPRT) activity and is partially involved in the cytotoxicity observed with Caco-2 cells [46]. Interestingly, VopT shows approximately 45% and 44% identity with the ADPRT domain of ExoT and ExoS of *P. aeruginosa*, respectively. ExoT and ExoS produced by *P. aeruginosa* both inactivating Rho family GTPases via GAP activity and are bifunctional proteins capable of interfering with other host signalling molecules via ADP ribosylation. In future experiments, we will elucidate the specific roles of T3SS-2 effector proteins in the cellular interaction of *V. parahaemolyticus* with marine protists.

In contrast to T3SS-2, homologues of the TTSS-1 genes are present in all *V. parahaemolyticus* strains examined as well as in some other *Vibrio* species, such as *V. harveyi*, *V. alginolyticus*, and *V. tubiashii* [21]. Although T3SS-1 is involved in its cytotoxicity of *V. parahaemolyticus* strain RIMD2210633 to HeLa cells [21], [41], we found that only the accessory T3SS-2 and not the T3SS-1 caused marine protists to lyse. There have been reports of other bacteria that possess two sets of T3SS, such as *E. coli*, *S. enterica* and members of the genera *Yersinia* and *Burkholderia* [48]. However, except for the case of the T3SSs in *Salmonella* spp., the differential roles of multiple T3SSs in a single bacterium have not yet been elucidated. In *S. enterica*, the first set of T3SS, SPI-1, mediates enterocyte invasion while SPI-2 influences survival within macrophages [48]. As we found no indication for the contribution of T3SS-1 to the fitness of *V. parahaemolyticus* strains interacting with a wide range of protist taxa, we propose that T3SS-1 may bear functions in the interaction with other marine eukaryotes.

A recent study on co-cultures of *V. parahaemolyticus* and the amoeba *A. castellanii* found no indication that survival of *V. parahaemolyticus* in the presence of amoebae is dependent on T3SS, TDH and quorum sensing [49]. However, the use of nutrient rich media (PYG; ATCC medium 712) rather than the marine minimal media used in the present study as well as the use of plate counts in combination with long-term incubation of 10–35 days may have lead to the contrasting results of the study by Laskowski-Arce and Orth. It could be that the experimental setup concealed cytotoxicity and that cryptic grazing and concomitant recycling of growth limiting nutrients contributed to the long-term survival of *V. parahaemolyticus*.

Like *V. cholerae*, *V. parahaemolyticus* is found in the free-living state as well as in association with particles. In a recent study on the interaction of *V. cholerae* with marine nanoflagellates we reported that biofilms are the protective niche enabling *V. cholerae* to survive protozoan grazing while their planktonic bacteria are eliminated [29], [50]. Grazing on planktonic *V. cholerae* was found to select for the biofilm-enhancing rugose variant, which is adapted to the surface-associated niche by the production of VPS exopolymers. Grazing resistance of *V. cholerae* biofilms was attained by exopolymer production and by the quorum sensing dependent secretion of antiprotozoal factors that inhibit protozoan feeding activity. Although the biofilm forming capacity of *V. parahaemolyticus* is well documented [35], we found no indication for biofilm-enhanced persistence or cytotoxicity of *V. parahaemolyticus* in the interaction with marine protists (Nouri *et al.*, unpublished data). Rather, our preliminary data indicate that T3SS-mediated cytotoxicity towards protists is reduced in biofilm-enhancing opaque variants of *V. parahaemolyticus* and is increased in opaR quorum sensing mutants.

A limited population of *V. parahaemolyticus* is capable of causing human diseases such as acute gastroenteritis. Almost all of the clinical *V. parahaemolyticus* isolates exhibit the Kanagawa phenomenon, a beta-type hemolysis on a special blood agar, and this phenomenon is caused by the thermostable direct hemolysin (TDH) [51]. Although a variety of *V. parahaemolyticus* serovars can cause human diseases, O3:K6 and a few other serotypes (O4:K68, O1:K25, and O1:KUT) have caused an increasing number of worldwide outbreaks of gastroenteritis since 1996 and are referred to as pandemic clonal group [52], [53], [11]. The prevalence of T3SS-2 positive and TDH positive genotypes among these pandemic clones and their isolation from both clinical and environmental samples suggest their involvement in environmental persistence and dissemination of *V. parahaemolyticus*. Our data show that T3SS-2 positive strains grow and persist better than T3SS-2 negative strains in the presence of environmental protists. The contribution of TDH is less clear. Although we found a small but significant difference of the *tdhAS* double-deletion mutant in co-culture with the nanoflagellate *C. roenbergensis*, plankton microcosm experiments of increased complexity did not show a significant effect of the *tdh* genotype for *V. parahaemolyticus* environmental invasiveness and persistence (data not shown).

An important factor for the global dissemination across marine environments is the organism's ability to invade local plankton communities. In microcosm experiments, we could demonstrate that T3SS-2 positive *V. parahaemolyticus* is able to invade coastal microbial communities faster by benefiting from protists as facultative hosts. Declines observed in the number of photoautrophic and heterotrophic protists suggest that both functional guilds are targeted by the *V. parahaemolyticus* T3SS-2. Studies from other bacteria interacting with higher eukaryotes suggest that T3SS target both animals and plant host cells [42], [43]. More mechanistic studies with representatives of marine phytoplankton are needed to unravel potential similarities of effector proteins and cytotoxicity between phototrophs and heterotrophs. Nevertheless, the present study illustrates the adaptive advantage of *V. parahaemolyticus* carrying the T3SS-2 encoding genome island VpaI-7. Global transportation via ballast water and aquaculture are thought to have driven the rapid dissemination of T3SS-2 and TDH positive *V. parahaemolyticus* pathotypes like the pandemic clone O3:K6 [11], [22]. Our experiments suggest that T3SS-2 positive *V. parahaemolyticus*, although underrepresented in coastal bacterioplankton communities, could persist and become periodically enriched when interacting with pelagic protists.

Cholera outbreaks are temporally related to phytoplankton blooms and have been hypothesized to result from enhanced availability of dissolved organic matter (DOM) for bacterial growth [54], [55]. Environmental surveys have demonstrated that massive increases in phytoplankton biomass (i.e. phytoplankton blooms) can result in enhanced growth of *V. cholerae* [28], [30]. In our experiments, artificial nutrient enrichment in combination with protist populations favored growth of T3SS-2 positive *V. parahaemolyticus*. *In situ* tracking of *V. parahaemolyticus* in mesocosm studies of increasing complexity should give better insight into processes and interactions in natural communities. Notably, T3SS-2 in *V. parahaemolyticus* shows similarity to the T3SS present in several pathogenic *V. cholerae* non-O1 and non-O139 isolates [56], which can cause gastroenteritis in humans. It will be interesting to test for the active roles of *V. cholerae* and other *Vibrio* spp. in the decline of phytoplankton populations and DOM release.

One hallmark in the ecology and epidemiology of pathogenic *Vibrio* spp. is the increased prevalence during warm summer months. The current model for the seasonal occurrence of cholera epidemics involves a number of abiotic factors favoring *V. cholerae* growth (e.g. temperature, pH, salinity, DOM) [55], [57]. The progressive spread of *V. parahaemolyticus* and its colonization of new areas has been related to an unusual increase in seawater temperatures in coastal zones [8]. A key issue in the epidemiology of *V. parahaemolyticus* is whether the emergence of O3:K6 isolates and their serovariants is the consequence of the effects of global warming. Several of the reported outbreaks, especially during the period 1996 to 1998, have been ascribed to elevated environmental temperatures and the El Nino phenomenon [10], [58], [59]. Our data indicate that increased water temperature as found in coastal waters accelerates the selective enrichment of T3SS-positive genotypes in the presence of aquatic protists.

Although much remains to be discovered, our results introduce the direct interaction of *V. parahaemolyticus* with aquatic protists as the functional context for eukaryote-targeting protein secretion machineries encoded on the pathogenicity island VPaI-7 and identify bacterial facultative parasitism as a mechanism that ensures the persistence and accumulation of *V. parahaemolyticus* in aquatic environments. We propose that *Vibrio*-protist interactions contribute to the selective enrichment of T3SS-2 positive *V. parahaemolyticus*, which may have important implications for seasonal population dynamics and the evolution of pathogenic clones. Recently, Persson *et al.* reported on the widespread occurrence of gene homologues of virulence-associated protein secretion machineries in a phylogenetically diverse set of genome sequenced marine bacteria and in the Global Ocean Sampling metagenomic databases [60]. Our experiments raise the question of how common and sophisticated facultative parasitism on protist populations is in aquatic bacteria – an interaction that may shape adaptation and biogeochemical fluxes in microbial food webs more than we currently appreciate.

## Supporting Information

Table S1 (XLS)
